# Cholecysto-hepatic duct serving as the only drainage pathway of bile from the intrahepatic to the extrahepatic biliary system in an infant: a case report

**DOI:** 10.1186/s12887-022-03491-z

**Published:** 2022-07-22

**Authors:** Samin Khoei, Payam Riahi Samani, Faezeh Fazelnia, Neda Pak

**Affiliations:** 1 Radiology resident, Imam Khomeini hospital complex, Tehran university of medical sciences, Tehran, Iran; 2 Radiology resident, Shariati hospital, Tehran university of medical sciences, Tehran, Iran; 3 Neda Pak, associate professor of radiology, children medical center of excellence, Tehran university of medical sciences (TUMS), Tehran, Iran

**Keywords:** Cholecystohepatic duct, Common hepatic duct agenesis, Jaundice, Case report

## Abstract

**Background:**

Cholecystohepatic duct is a rare anomaly of the biliary system which involves drainage of bile into the gallbladder which may be associated with agenesis of the common hepatic duct or common bile duct.

**Case presentation:**

A 2.5-month-old infant presented to our emergency department with icterus. He had a history of esophageal atresia and imperforate anus which had been treated surgically by thoracotomy, esophagostomy, gastrostomy and colostomy placement. Following imaging studies by ultrasound and MRCP, the diagnosis of common hepatic duct agenesis was made. Cholecystohepatic duct was present as the solitary drainage pathway of bile from the intrahepatic to extrahepatic biliary system.

**Conclusions:**

Cholecystohepatic ducts need a high index of suspicion to be diagnosed on preoperative hepatobiliary imaging. As they may be asymptomatic, they are predisposed to iatrogenic injury during hepatobiliary surgeries.

## Background

Aberrancies in the biliary ductal system is defined as bile ducts that directly drain a portion of the liver into the extrahepatic biliary system, cystic duct, or gallbladder, the latter being specified as a cholecystohepatic duct; a very rare malformation with few cases reported in the literature [1, 2]. 

Here we present a case of common hepatic duct agenesis with cholecystohepatic duct as the solitary drainage pathway of intrahepatic bile to gallbladder and then extrahepatic duct in an infant with other malformations.

## Case presentation

A 2.5-month-old boy presented to our emergency room with icterus for the previous two days. He had a history of esophageal atresia and imperforate anus, that had been treated with thoracotomy, esophagostomy, gastrostomy and colostomy placement on the second day of his life. No associated symptoms such as fever or respiratory distress existed upon presentation.

On physical examination, sclera icterus and stool leakage from colostomy were the only positive findings. The infant appeared otherwise normal.

The laboratory examination revealed elevated liver enzymes (Alkaline phosphatase: 836IU/L, Gamma-GT: 1020IU/L, Aspartate aminotransferase: 201IU/L, and Alanine aminotransferase: 124IU/L) and direct hyper-bilirubinemia (total bilirubin: 9.7 mg/dl and direct bilirubin: 5.8 mg/dl). Klebsiella pneumonia was isolated from the colostomy culture.

Left-sided SVC, mild tricuspid regurgitation, and mild pulmonary insufficiency were noted on echocardiography.

**Fig. 1:**
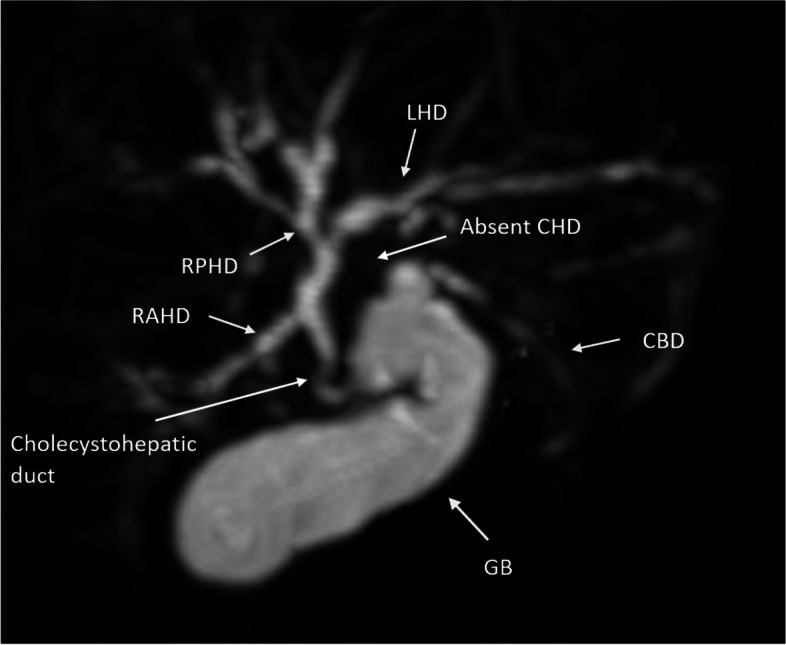
MRCP image shows no common hepatic duct (CHD) in its anatomic location and there is a duct connecting RAHD to neck of gallbladder (GB) as cholecystohepatic duct. Trifurcation of the right anterior (RAHD), right posterior (RPHD), and left hepatic duct (LHD) is visualized. Diameter of CBD is normal. Intrahepatic bile ducts are mildly dilated according to the patient’s age

On abdominopelvic ultrasound mildly dilated central with normal-sized peripheral intra-hepatic biliary ducts were visualized. The proximal cystic duct (5 mm) appeared dilated and contained a sludge ball within. Layering sludge inside the gallbladder and common bile duct was seen. Otherwise, the exam was unremarkable considering abnormal ultrasound findings with suspicion of biliary tree anomaly, Magnetic resonance cholangiopancreatography (MRCP) was performed which revealed trifurcation of the right anterior, right posterior (RPD), and left hepatic duct (LHD) anterior to the portal venous bifurcation. The intrahepatic biliary ducts appeared mildly dilated. Common hepatic duct was not visible in the expected location after the confluence of RAD, RPD, and LHD. Instead, an aberrant cholecystohepatic duct was noted as the only connection draining bile from the right anterior hepatic duct towards neck of the gallbladder. A mildly dilated cystic duct drained bile from the gallbladder into CBD, which was normally tapered until termination in the ampulla of Vater. (Fig. 1)

The patient was treated with Cefotaxime and Amikacin due to positive colostomy culture and Ursodeoxycholic acid. The symptoms and laboratory data improved, and the patient was discharged in seven days.

He remained symptom-free in the next 6 months when he was recalled for the final surgery at which colon interposition was performed and during this surgery, portahepatis was explored as well, no fibroproliferative tissue was present at the anatomic site of CHD indicating biliary agenesis rather than atresia. The post-operative days were uneventful, and he was discharged with no symptoms, including icterus.

Concerning the patient’s biliary anomaly, and as he remained symptom-free on medical treatment (Ursodeoxycholic acid), close follow-up was preferred upon surgery. He did well in the next 15 months and is yet to be followed.

## Discussion and conclusion

Anatomical Variations or anomalies in the biliary system are not rare, involving gallbladder and extra and intrahepatic bile ducts. These variations may be clinically silent but are important to the surgeons to prevent biliary complications during liver transplantations, hepatobiliary and gallbladder surgeries [3, 4]. These anatomical variations are becoming more recognized preoperatively by the advantage of MRCP, which is mostly performed before liver transplantation, partial hepatectomy, or in suspected cases of biliary tree anomalies and choledochal cysts.

The normal most frequent biliary anatomy is as follows: Peripheral intrahepatic bile ducts start in the center of hepatic lobules, run along the portal venous and hepatic arterial branches towards the center, and successively merge to form the sub-segmental and segmental hepatic branches. The segmental ducts of second and third (lateral segmental branches) and fourth (medial segmental branch) hepatic segments form the left hepatic duct (LHD), the fifth and eighth segmental ducts together form the right anterior [5] or ventrocranial duct, and the sixth and seventh segmental ducts form the right posterior (RPD) or dorsocaudal duct [4]. The RAD and RPD then merge together as the right hepatic duct (RHD). RHD and LHD join and form the common hepatic duct (CHD) anterior to the portal venous bifurcation. The cystic duct -from the gallbladder- unites with the lateral side of the CHD in the middle third of the way between the origin of CHD and ampulla of Vater, and from now on, the common bile duct forms (CBD). The abovementioned anatomy is the case in 58-62.6% of people [4]. 

The biliary tree anomalies/variations which are most prevalent in the right ductal system include aberrant/accessory bile ducts, biliary cysts, anomalies associated with the anomalous situs, and pancreaticobiliary junction anomaly [4]. We review the first group in the current article:

Aberrant duct is present when a duct runs in a variant route other than the normal, and that is the only duct draining that particular segment, but accessory duct is the case when a duct is a supernumerary one added to the normal duct draining the segment. These anomalies, in the order of prevalence, include: (1) Drainage of the RPD to LHD, CHD, or CD (the crossover anomaly). (17-18.4%), (2) The hepatic ductal trifurcation, where the RPD, RAD and LHD merge in a single point (5–19%), (3) Drainage of the RHD to the CD or CHD, (4) The cystic duct insertion to the medial wall of the CHD, which is extremely important to know upon the cholecystectomy procedure, (10–18%) (5) The CD insertion to the distal third of the common duct which is called the CD low insertion (8–11%), (6) A parallel course of the CD in close approximation with the CHD for more than 2 cm, which is called the long parallel course, (7.5%) (7) Insertion of the CD to the RHD, high insertion of the CD to CHD, double CD and short CD, that are noted in rare cases, [4] (8) Subvesical bile ducts or cholecystohepatic ducts, which we discuss in greater detail in the following paragraph, are the ones which drain from the right hepatic segments directly into the gallbladder or the CD.

Cholecystohepatic duct is an uncommon anomaly of the biliary system and occurs in 0.7–1.2% of the population [1, 2].  Only 29 cases of cholecystohepatic duct with agenesis of CBD/CHD are reported in the literature since 1945 [5–7].  In this rare anomaly, bile drains into the gallbladder which is connected to duodenum via cystic duct with agenesis of the CBD or CHD [8].

Pathogenesis is not well understood but it is hypothesized to be related to failure of normal development of the biliary system from the cystic diverticula: failure of recanalization, [9] or delayed division of the hepatic antrum to cystic diverticulum and hepatic diverticulum [10].

Absence of the hepatic duct in the presence of an alternate anomalous and thus far adequate drainage pathway, suggests that the development of the hepatic duct may be partly dependent on bile flow at some point during embryogenesis. The apparently effective and very rare alternate path in this case might have facilitated a flow-related steal leading to complete developmental failure of the hepatic duct. An alternative idea is that primary absence of a hepatic duct facilitated the development of the rather large alternate pathway which is a less possible hypothesis as daily bile flow is thought to be low in volume until birth and normal feeding is established.

This anomaly is categorized into five types: Type I involves drainage of the right and left hepatic ducts separately into the gallbladder without presence of CHD. In Type II, the left and right hepatic ducts join together just before entrance to the gallbladder, with no obvious formation of CHD. In Type III, right and left hepatic ducts join and form CHD and then enter the gallbladder. CHD may enter the superior wall of gallbladder (IIIA), neck of gallbladder (IIIB), posterior wall of gallbladder (IIIC); and the fundus (IIID). And finally, in type IV several small ducts drain bile from all hepatic segments to the gallbladder [8].

Among all reported cases, seven cases are classified as type I, one case as type II,16 ones as type III (four cases IIIa, nine cases IIIb, one case IIIc, and two cases IIId), and two cases as type IV. One case was a variant of type I, in which only RHD drained into gallbladder, and left duct drained into duodenum. Information about two of the cases were not available to mention type of the anomaly [6].

Some of them were completely asymptomatic with no abnormality in laboratory data and diagnosed in autopsy or gall bladder surgeries (which surprised the surgeons and changed the simple cholecystectomy to a more complicated surgery like hepaticojejunostomy), and some others were symptomatic and presented with abdominal pain, jaundice, nausea and vomiting, cholangitis, symptomatic gall stone, or cholecystitis [6].

All of them except three cases were diagnosed as having this anomaly in adulthood. (ranging from 22 to 78 years old) Adult cases were reported with no associated anomaly, while two of the pediatric cases had esophageal atresia and Meckel’s diverticula; one of them had also other anomalies such as dysplastic thumb, absent uterus, and vaginal atresia, with normal chromosomic study [11]. All three pediatrics were diagnosed as having cholecystohepatic duct at age of 3–4 years following episodes of jaundice [11, 12].

None of the 29 cases were diagnosed preoperatively with ultrasound, and some of them were even misdiagnosed as having choledochal cyst [11].  Most common ultrasound findings were distended gallbladder, gallbladder stones, chronic cholecystitis, and dilated CBD or intrahepatic bile ducts.

Most cases (19 of 29 cases) were diagnosed during open exploration (most of them also underwent intraoperative cholangiography). Some were diagnosed following complications of cholecystectomy. Two cases were diagnosed in post-mortem autopsy [6]. In none of them, MRCP was mentioned to play a role in diagnosis.

Main surgical management involved partial cholecystectomy and choledochoplasty, Roux-en-Y hepaticojejunostomy with the RPD or anastomosis with the cystic duct, cholecystoduodenostomy, primary anastomosis and biliary reconstruction, and closure of the duct if the drainage area was small [1, 6]. 

Our patient is another example of cholecystohepatic duct serving as the only drainage pathway from intrahepatic ducts toward the gallbladder, which is a unique case from several perspectives. He is the youngest case to be diagnosed as having this very rare anomaly at the age of only 2.5 months old. On the other hand, he was diagnosed with MRCP, while previously reported cases were diagnosed with invasive methods. MRCP revealed trifurcation of RAHD, RPHD, and LHD without presence of CHD in its typical position and cholecystohepatic duct connecting RAHD to neck of gallbladder. According to the abovementioned classification of cholecystohepatic ducts, it cannot be confined to any of the five groups as the bile is drained via RAHD to gallbladder neck. He had other anomalies such as esophageal atresia and imperforate anus. Surprisingly, among three previously reported pediatric cases, two of them had esophageal atresia as well, which raises the suspicion that this bile duct anomaly could have association with esophageal atresia that may remain undiagnosed since patients could be totally asymptomatic. Interestingly, esophageal atresia and imperforate anus have association with an important biliary tract disease, that is biliary atresia; [13] which was the initial suspected diagnosis of our patient when he referred with jaundice.

Our patient experienced one episode of jaundice during systemic infection and sepsis which subsided with medical treatment. His direct hyperbilirubinemia could either be due to sepsis itself or due to inadequacy of the anomalous pathway under stress. On 15-month follow-up of the patient, he has remained asymptomatic with no other episode of jaundice, which suggests that jaundice could more likely be due to sepsis rather than inadequacy of the biliary pathway. However, long-term follow-up would provide more information. Concerning the mild symptoms at presentation, relief of them with medical treatment and simultaneous major surgical procedures done for repair of the concomitant esophageal atresia and imperforate anus; medical treatment and watchful waiting were preferred for his management. What is important in these cases with absent extrahepatic ducts, is to differentiate between biliary atresia and biliary agenesis. Biliary atresia results from an inflammatory and fibrosing obstruction and can be diagnosed during surgery by visualization of fibroproliferative tissue at porta hepatis. On preoperative ultrasound or MRCP, absent or small gallbladder (≤ 15 mm in length), irregular gallbladder wall, and triangular cord sign (tubular or triangular echogenic cord in ultrasound imaging at porta hepatis representing fibrous ductal remnant of the extrahepatic bile duct) are notable [14, 15]. But there is a subtype of biliary atresia known as “embryonic biliary atresia”, in which extrahepatic bile ducts might be absent due to early injury of bile ducts during fetal development without any fibroproliferative tissue detectable in surgery [16].  So differentiation of biliary atresia from biliary agenesis may be difficult in these cases. In biliary atresia, patients usually present with icterus in neonatal period with development of liver cirrhosis if left untreated; this is a clinical presentation different from our patient’s. And also, the presence of dilated intrahepatic ducts in our case is a finding against biliary atresia [17].  Therefore, we believe our patient is a case of biliary agenesis rather than atresia.

Considering the fact that cholecystohepatic ducts may be completely silent, there should be a high index of awareness of this anomaly to be correctly diagnosed. Underutilization of preoperative imaging or unawareness by surgeons may predispose to inappropriate decisions or iatrogenic injury in patients with pancreaticobiliary tract anomalies. We recommend MRCP to be performed prior to hepatobiliary surgeries in pediatric patients with icterus and any sign of biliary abnormality on ultrasound other than typical findings for biliary atresia, especially in cases with concomitant extrahepatic anomalies. Being aware of this rare anomaly, surgeons would perform a careful exploration of the extrahepatic biliary anatomy before cholecystectomy surgeries.

## Data Availability

Derived data supporting the findings of this study are available from the corresponding author on request.

## Abbreviations

SVC: Superior vena cava
RAD: Right anterior duct
RPD: Right posterior duct
LHD: Left hepatic duct
CHD: Common hepatic duct
CBD: Common bile duct
MRCP: Magnetic resonance cholangiopancreatography
